# A Scale to Assess the Methodological Quality of Studies Assessing Usability of Electronic Health Products and Services: Delphi Study Followed by Validity and Reliability Testing

**DOI:** 10.2196/14829

**Published:** 2019-11-15

**Authors:** Anabela G Silva, Patrícia Simões, Rita Santos, Alexandra Queirós, Nelson P Rocha, Mário Rodrigues

**Affiliations:** 1 School of Health Sciences University of Aveiro Aveiro Portugal; 2 Higher School of Technology and Management of Águeda Aveiro Portugal; 3 Department of Medical Sciences University of Aveiro Aveiro Portugal

**Keywords:** quality of health care, eHealth, mHealth, efficiency

## Abstract

**Background:**

The usability of electronic health (eHealth) and mobile health apps is of paramount importance as it impacts the quality of care. Methodological quality assessment is a common practice in the field of health for different designs and types of studies. However, we were unable to find a scale to assess the methodological quality of studies on the usability of eHealth products or services.

**Objective:**

This study aimed to develop a scale to assess the methodological quality of studies assessing usability of mobile apps and to perform a preliminary analysis of of the scale’s feasibility, reliability, and construct validity on studies assessing usability of mobile apps, measuring aspects of physical activity.

**Methods:**

A 3-round Delphi panel was used to generate a pool of items considered important when assessing the quality of studies on the usability of mobile apps. These items were used to write the scale and the guide to assist its use. The scale was then used to assess the quality of studies on usability of mobile apps for physical activity, and it assessed in terms of feasibility, interrater reliability, and construct validity.

**Results:**

A total of 25 experts participated in the Delphi panel, and a 15-item scale was developed. This scale was shown to be feasible (time of application mean 13.10 [SD 2.59] min), reliable (intraclass correlation coefficient=0.81; 95% CI 0.55-0.93), and able to discriminate between low- and high-quality studies (high quality: mean 9.22 [SD 0.36]; low quality: mean 6.86 [SD 0.80]; *P*=.01).

**Conclusions:**

The scale that was developed can be used both to assess the methodological quality of usability studies and to inform its planning.

## Introduction

### Background

Methodological quality can be defined as “the extent to which study authors conducted their research to the highest possible standards” [1]. It should be considered both when interpreting individual study findings and when conducting systematic reviews and aggregating findings from different studies and making recommendations [1,2]. However, the critical assessment of the quality of studies is a complex process that must consider several different aspects of the study, which may vary depending on the type of study and on the subject of research [1,3,4]. Therefore, this process is usually performed with the aid of critical appraisal tools previously developed for that specific purpose. This is common practice in the field of health, where a number of critical appraisal tools exist to assist the assessment of the methodological quality of studies [1,3-5]. There are different tools depending, for example, on whether studies are randomized clinical trials aiming to assess the effectiveness of interventions [5] or assess the validity and/or reliability of measurement instruments [6] or are diagnostic accuracy studies [3]. However, we were unable to find any critical tool to guide the assessment of methodological quality of usability studies, neither for electronic health (eHealth) applications nor for general applications.

According to the International Standards Organization 9241-11, usability refers to the “extent to which a system, product or service can be used by specified users to achieve specified goals with effectiveness, efficiency and satisfaction in a specified context of use” [7]. Therefore, usability evaluation is an important part of the process of development of any system, product, or service [8] and can be formative or summative, that is, its main focus may be to detect and solve problems or to meet the metrics associated with the system, product, or service task and goals [9]. The complex nature of usability often requires the use of combined approaches for its assessment [8], involving, for example, the triangulation of methods, the use of both experts and end users, and different settings (eg, laboratory or real context). Furthermore, the type of instruments and procedures that are more adequate depend on several factors, such as the aim of the usability assessment (formative or summative) and on the development phase of the system, product, or service [10]. A methodologically sound assessment of usability is crucial to minimize the probability of errors and undesirable consequences and to increase the probability of use by a large proportion of the target end users [7]. In the field of health, usability contributes to enhance patient safety and quality of care, and recommendations aiming to enhance these by means of improving the usability have been published [11] as well as protocols to measure and validate user performance before deployment [11]. However, poor assessment of usability is common practice and impacts the quality of the eHealth apps [11]. Therefore, having a reference guide that could be used both to inform the design of usability studies and to assess the methodological quality of published studies is of paramount importance and constitutes a step forward in the field of usability.

### Objectives

The aims of this study were to develop a scale to assess the methodological quality of studies assessing usability of mobile apps and to perform a preliminary analysis of its feasibility, reliability, and construct validity on studies assessing usability of mobile apps, measuring aspects of physical activity.

## Methods

**Figure 1:**
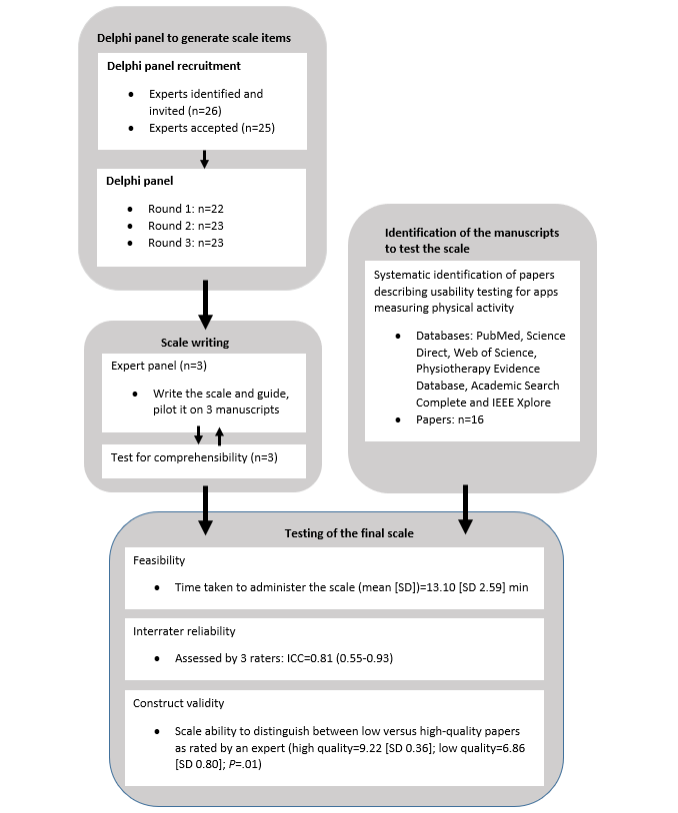
Flowchart of study procedures. ICC: intraclass correlation coefficient.

This study comprised 3 phases: (1) a 3-round Delphi panel to generate a pool of items considered important when assessing the quality of studies on usability; (2) a panel of experts to write the scale and the guide to assist in the use of the scale when assessing the quality of studies on usability; (3) testing of the developed scale, including the assessment of feasibility, interrater reliability, and construct validity. Figure 1 shows the flow of procedures for this study.

### Delphi Panel

The Delphi method was used because it is recommended to determine the consensus for a predefined problem when there is little information, and one must rely on the opinion of experts. It is a structured multistage process to collect information from an expert panel about a certain topic to reach consensus based on structured group communication [12].

#### Expert Selection

To take part in the study, experts had to meet the following criteria: (1) have experience conducting studies on usability and (2) have previous published work on assessment of usability. In addition, we aimed to recruit participants with diverse academic backgrounds (Technology, Health, and Design) so that different points of view could be gathered. Participants who complied with these criteria were identified by team members. A sample size of at least 20 experts has been suggested as appropriate [12,13]. To account for potential dropouts through the rounds, a total of 26 experts were invited to enter the study by an individual email or a phone call, and 25 of them accepted. Experts’ anonymity was maintained throughout the study.

#### Development of Consensus

This Delphi study was organized in 3 rounds. In the first round, participants were sent an email explaining the study with a link to a questionnaire developed using Google Forms and were asked to identify, by order of relevance, a minimum of 4 items that they thought were important to consider when assessing the quality of studies on usability. They were also asked to provide a justification for their choice.

The results of the first round were collated and then grouped into categories and subcategories with the same meaning so that at the end of this process, all items identified by all experts were allocated to a subcategory. This process was performed independently by 3 researchers (AGS, PS, and AR), who then met to compare their coding, and a consensus was reached. Each subcategory gave origin to a statement about an aspect that should be checked when assessing usability studies’ methodological quality. The list of statements was sent back to experts in the second round. In this round, experts were asked to rate the relevance of each statement using a 9-item Likert scale (1—item not important to 9—item very important). Participants were also asked to give their opinion on the formulation of the items. Consensus on the inclusion of 1 item in the scale was considered when 70% or more participants scored the item as 7 to 9 and less than 15% of participants scored it as 1 to 3. Consensus on the exclusion of 1 item was considered when 70% or more participants scored the item as 1 to 3 and less than 15% of participants scored the item as 7 to 9 [14,15]. The changes recommended by experts on the writing of each item, and which were considered relevant, were included in the third round.

In the third round, each expert has been presented with his/her previous score of each item and the ratings of the remaining experts summarized as absolute frequencies and presented in a graphic format. Experts were then asked whether they would like to reconsider their previous rating. The final list included all items that were classified with 7 to 9 regarding the degree of importance by at least 70% of the participants [14].

For each round, a minimum response rate of 70% was required to consider the round valid [16]. Experts had between 2 and 3 weeks to respond to each round, and reminders were sent at the end of the first and second weeks to those that had yet to reply.

#### Panel of Experts for Scale Writing

A total of 3 researchers (AGS, ARS, and PS) met to agree on the final writing of the scale to assess the quality of studies evaluating usability and how they should be ordered and prepared a first draft of the guide/manual of use to assist on using the scale. It was decided that an item should be scored as 0 if it was not assessed or not described in the study being appraised and as 1 if the item was assessed and that adding up the individual item score would result in a final global score. This first draft was piloted independently by the researchers on 3 manuscripts. In the second meeting, the panel revised the first draft of the scale based on their experience of using it. As it was decided that 2 items of the scale could be considered *not applicable*, we determined that the final score should be presented as percentage (ie, [number of items scored 1/total numbers of items applicable] × 100). This version of the scale was then sent to 3 experts external to this panel for comprehensibility assessment. These experts made only minor suggestions that were considered for inclusion, and the final version of the scale was named as Critical Assessment of Usability Studies Scale (CAUSS).

### Feasibility, Interrater Reliability, and Construct Validity of the Final Scale

#### Feasibility and Interrater Reliability

To evaluate the feasibility and interrater reliability of the CAUSS, a systematic search for studies assessing usability of mobile apps measuring aspects of physical activity was performed on PubMed, Science Direct, Web of Science, Physiotherapy Evidence Database, Academic Search Complete, and IEEE Xplore using a combination of the following expressions: physical activity, mobile applications, and usability. All databases were searched since January 1, 2000, and the search was performed on October 29 and 30, 2017. We chose to use studies on the usability of mobile apps measuring aspects of physical activity to assess reliability as this study was conducted within the context of a research project on mobile (ie, eHealth or mobile health [mHealth]) apps to promote physical activity. To be included in the reliability part of this study, manuscripts had to (1) be full text; (2) specify the assessment of usability as one of its aims; and (3) describe the assessment of usability of an eHealth or mHealth app aiming primarily at measuring physical activity at any stage of development. A total of 16 studies met the inclusion criteria [17-32]. These studies were assessed independently by 3 authors (AGS, ARS, and PS) using the CAUSS and the respective guide. An intraclass correlation coefficient (ICC; 2-way random; absolute agreement) was used to compare the total score among raters, and an ICC of at least 0.7 was considered acceptable [33]. In addition, a repeated measures analysis of variance was also used to explore for significant differences between the scores of the 3 raters.

Feasibility was evaluated by assessing the time taken to assess the 16 studies using the CAUSS.

#### Construct Validity of the Scale

As there was no gold standard against which to compare the results of our scale, construct validity was assessed using a method adapted from Jadad et al [34] and Yates et al [35]. Articles were allocated to a group by a rater with extensive knowledge on both usability and methodological quality (NPR). This rater categorized each one of the 16 articles as low or high quality. Construct validity of the scale was assessed by testing whether it was able to discriminate between these categories. The consensus ratings of the 3 judges (AGS, ARS, and PS) who assessed each manuscript using the CAUSS (as detailed in the reliability section) were used for this analysis. A Student *t* test (data followed a normal distribution) was used to compare the scores of the articles classified as low and high quality.

## Results

### Delphi Panel

Of the 25 experts that entered the study, 11 (44%) were females, 21 (84%) held a Doctor of Philosophy degree, and their areas of academic background were diverse (Sciences and Technology of Communication, Engineering and Mathematics, Health, and Design; Table 1).

The first round was completed between April and June 2018, and a total of 22 out of 25 experts (88%) answered the questionnaire. In this round, the panel of experts generated a total of 121 statements where each person generated 5 statements on average (SD 0.87). The total statements were grouped in 22 main topics (Table 2), of which 6 were excluded by 3 members of the research team (Textbox 1) because they were not specific of usability studies and/or because they were out of scope (ie, not related to usability). The remaining topics were transformed into 15 questions and sent back to experts in round 2 (Table 2). Round 2 was completed by experts between November 2018 and January 2019. A total of 23 experts (92%) answered the questionnaire. Of the 15 questions identified, 13 reached consensus for inclusion.

The third and final round was completed between January and February 2019 by 23 out of 25 experts (92%). In this round, 14 of the 15 statements reached consensus and were included in the scale. However, the statement that did not reach consensus was also included because most of the experts (18/23, 78%) classified it with 6 or more out of a maximum score of 9. Table 3 shows the score of the 15 questions after rounds 2 and 3. These final statements were then used by the panel of 3 experts to write the final scale and its guide/manual of use (Multimedia Appendix 1).

**Table 1:** Characterization of experts participating in the Delphi panel (n=25).

Characteristics		Values
**Gender, n (%)**		
	Male	14 (56)
	Female	11 (44)
Age (years), median (IQR^a^)		42 (14)
**Education, n (%)**		
	Masters	4 (16)
	Doctoral	21 (84)
**Academic background, n (%)**		
	Sciences and Technology of Communication	7 (28)
	Engineering and Mathematics	11 (44)
	Health	5 (20)
	Design	2 (8)
**Current professional occupation, n (%)**		
	University lecturer	16 (64)
	Researcher	8 (32)
	Designer	1 (4)
Experience in usability assessment (years), median (IQR)		10 (10)

^a^IQR: interquartile range.

**Table 2:** Subcategories generated after round 1 and included in round 2.

Subcategories	Questions sent back to experts in round 2
Valid measurement instruments	Did the study use valid measurement instruments of usability (ie, there is evidence that the instruments used assess usability)?
Reliable measurement instruments	Did the study use reliable measurement instruments of usability (ie, there is evidence that the instruments used have similar results in repeated measurements in similar circumstances)?
Procedures adequate to the study’s objectives	Was there coherence between the procedures used to assess usability (eg, instruments and context) and study aims?
Procedures adequate to the development stage of the product	Did the study use procedures of assessment for usability that were adequate to the development stage of the product/service?
Procedures adequate to the participants’ characteristics	Did the study use procedures of assessment for usability adequate to study participants’ characteristics (eg, children and elderly require different instruments)?
Triangulation	Did the study employ triangulation of methods for the assessment of usability?
Combination of users’ and experts’ evaluation	Was usability assessed using both potential users and experts?
Experience of the investigator that conducted the usability evaluation	Was the investigator that conducted usability assessments adequately trained?
Investigator conducting usability assessment external to the development of the product/service	Was the investigator that conducted usability assessments external to the process of product/service development?
Assessment in real context or close to real context	Was the usability assessment conducted in the real context or close to the real context where product/service is going to be used?
Number of participants (potential users and/or experts)	Was the number of participants used to assess usability adequate (whether potential users or experts)?
Representativeness of participants (potential users and/or experts)	Were participants who assessed the product/service usability representative of the experts’ population and/or of the potential users’ population?
Representativeness of the tasks to perform on the usability evaluation	Were the tasks that serve as the base for the usability assessment representative of the functionalities of the product/service?
Continuous and prolonged use of the product	Was the usability assessment based on continuous and prolonged use of the product/service over time?
Analysis of the results	Was the type of analysis adequate to the study’s aims and variables assessed?

**Subcategories generated after round 1 and not included in round 2.**

1. Compliance with ethical principles
2. Pilot study before the main study
3. Definition of a protocol before study beginning
4. Description of study objectives, tasks, methods, measurement instruments, measures, context, and mobile app
5. The study is possible to replicate and/or reproduce
6. Others (negative impacts of usability, sample motivation, and development cycle)

**Table 3:** Results from rounds 2 and 3 of the Delphi panel.

Questions	Second round score, n (%)			Third round score, n (%)			Consensus
	1-3	4-6	7-9	1-3	4-6	7-9	
Did the study use valid measurement instruments of usability (ie, there is evidence that the instruments used assess usability)?	0 (0)	0 (0)	23 (100)	0 (0)	0 (0)	23 (100)	Yes
Did the study use reliable measurement instruments of usability (ie, there is evidence that the instruments used have similar results in repeated measurements in similar circumstances)?	0 (0)	3 (13)	20 (87)	0 (0)	0 (0)	23 (100)	Yes
Was there coherence between the procedures used to assess usability (eg, instruments, context) and study aims?	0 (0)	4 (17)	19 (83)	0 (0)	2 (9)	21 (91)	Yes
Did the study use procedures of assessment for usability that were adequate to the development stage of the product/service?	0 (0)	5 (22)	18 (78)	0 (0)	4 (17)	19 (83)	Yes
Did the study use procedures of assessment for usability adequate to study participants’ characteristics (eg, children and elderly require different instruments)?	0 (0)	2 (9)	21 (91)	0 (0)	1 (4)	22 (96)	Yes
Did the study employ triangulation of methods for the assessment of usability?	0 (0)	5 (22)	18 (78)	0 (0)	6 (26)	17 (74)	Yes
Was usability assessed using both potential users and experts?	0 (0)	5 (22)	18 (78)	0 (0)	4 (17)	19 (83)	Yes
Were participants who assessed the product/service usability representative of the experts’ population and/or of the potential users’ population?	0 (0)	3 (13)	20 (87)	0 (0)	2 (9)	21 (91)	Yes
Was the investigator that conducted usability assessments adequately trained?	1 (4)	4 (17)	18 (78)	1 (4)	1 (4)	21 (91)	Yes
Was the investigator that conducted usability assessments external to the process of product/service development?	1 (4)	10 (44)	12 (52)	1 (4)	10 (43)	12 (52)	No^a^
Was the usability assessment conducted in the real context or close to the real context where product/service is going to be used?	0 (0)	5 (22)	18 (78)	0 (0)	2 (9)	21 (91)	Yes
Was the number of participants used to assess usability adequate (whether potential users or experts)?	0 (0)	2 (9)	21 (91)	0 (0)	0 (0)	23 (100)	Yes
Were the tasks that serve as the base for the usability assessment representative of the functionalities of the product/service?	0 (0)	0 (0)	23 (100)	0 (0)	0 (0)	23 (100)	Yes
Was the usability assessment based on continuous and prolonged use of the product/service over time?	0 (0)	9 (39)	14 (61)	0 (0)	6 (26)	17 (74)	Yes
Was the type of analysis adequate to the study’s aims and variables assessed?	0 (0)	1 (4)	22 (96)	0 (0)	0 (0)	23 (100)	Yes

^a^This item was included because most of the experts (n=18, 78%) classified it with 6 or more out of a maximum score of 9.

### Feasibility, Interrater Reliability, and Construct Validity of the Final Scale

#### Feasibility

The time taken (in minutes) to assess the articles using the scale varied between 10 and 18 min (mean 13.10 [SD 2.59] min).

#### Interrater Reliability

The 3 judges assessing the interrater reliability achieved an ICC of 0.81 (0.55-0.93) for the total scoring. Mean (SD) for the 3 raters was 8.63 (1.41), 8.60 (2.00), and 8.44 (1.50), and no significant difference was found between them (*F*_2,14_=0.29; *P*=.75). Multimedia Appendix 2 presents the raters’ score for each of the 15 items of the scale.

#### Construct Validity

The rater classified 9 articles as high quality and 7 articles as low quality. Mean (SD) of the scale’s total score for the 2 groups of articles using the consensus score for each paper was significantly different: 9.22 (0.36) for the high-quality group and 6.86 (0.80) for the low-quality group (*P*=.01).

## Discussion

This study presents a scale to assess the methodological quality of studies assessing usability, which was developed through a modified Delphi panel. Results of a pilot test of the scale on papers assessing usability of eHealth apps that measure physical activity suggest that the scale is feasible, valid, and reliable.

### Validity

Content validity of the scale is supported by the consensus generated among a group of experts with diverse backgrounds and areas of expertise allowing us to capture a broad perspective on usability [32]. In addition, many of the methodological aspects of usability studies covered in the 15 items of the scale have been previously reported as relevant, such as validity and reliability of the instruments used to assess usability, adequate sample size [9,10], combined use of different methods of usability assessment, and adequacy of study procedures to the development stage of the product/service [36].

Further evidence on the scale validity comes from the fact that general items such as reliability and validity of instruments used, adequate sample size, competence of the assessor, appropriateness of analysis methods, or representativeness of participants are also reported in other scales [3,4,6,34] and from the scale’s ability to distinguish between low- and high-quality trials (construct validity).

### Reliability

The interrater reliability was acceptable, but the lower limit of the confidence interval is below the cut off for acceptable reliability. The raters involved in reliability testing had diverse backgrounds (health and engineering) and different degrees of experience rating the methodological quality of studies, which may have had an impact on the reliability results. Items 6, 7, 9, and 13 were the items of the scale where disagreement was more frequent. The lack of detail of the Methods section of the papers assessed and the different degrees of expertise of the raters on quantitative and qualitative data analysis may help explain why disagreement was more marked for these items. Furthermore, and for item 9 (participants representative of the experts’ population and/or of the potential users’ population), a few questions arose during the discussion to reach consensus among the 3 raters, particularly regarding the minimal set of characteristics that study authors need to provide to allow the reader/assessor to be able to judge on whether study participants were representative. For example, the users’ age, sex, and previous experience using mobile phones and apps are important aspects to consider when judging the representativeness of the sample [36]. Similarly, a low ratio between the initial number of participants invited and those that entered the study as well as a less optimal recruitment process can lead to a more homogeneous sample with specific characteristics, which is less likely to be representative of the wider population [37]. For experts, area of expertise, years of practice, and previous experience using similar applications are examples of relevant characteristics to consider. However, a clear and complete description of participants, either experts or potential users, was generally not given in the studies assessed. These aspects may have contributed to the lower agreement on the referred items.

In contrast, items 8 (use of both potential users and experts), 11 (was the investigator that conducted usability assessments external), and 15 (continuous and prolonged use of the product/service) were consensual for all studies. Interestingly, all studies (except one) received the same rating for items 8 and 9 (insufficient information provided by study authors/no) and 15 (yes). The apparent higher objectivity of these items, the absence of information on who was the person conducting usability assessments, and the clear description of the period during which the application was used may explain the higher agreement between raters for these items.

### Identified Shortcomings of the Papers Assessed

There were several aspects that were consistently not considered or for which insufficient detail was provided by authors of the papers assessed using our scale. Reliability and validity of the instruments used were never reported in the papers assessed. When this item was rated as “yes,” meaning that studies employed reliable and/or valid instruments, it was because the instruments used were known to be valid and reliable. For data collected using qualitative methodologies, there was insufficient detail on how the analysis was conducted and how many researchers were involved. Using valid instruments (ie, instruments that measure what they are expected to measure) and instruments that are reliable (ie, instruments that give consistent ratings in the same conditions) are fundamental so that one can trust on the results of the assessment [38]. Information regarding who was the person performing usability assessments and previous experience and/or training to perform usability assessment was seldom given. However, previous experience or adequate training is fundamental, particularly for qualitative assessments of usability, and having an interest on the service/product being tested may bias the results. This has been shown in the field of health whether beliefs and expectations have been found to have an impact on the study results [39]. The lack of clarity of reports on usability assessment has already been pointed by other authors [40]. Future studies assessing usability of products/services should clearly report on these details. Poor reporting may reflect the lack of planning and poor methodological quality.

### Limitations

The underlying assumption of calculating the total score of CAUSS by simply adding individual items is that all items are equally important to the final score. This is the simplest and most commonly used solution, but it does not account for the varying relevance of individual items to the construct being measured [41]. In contrast, adding up items makes the scale easier to score and, potentially, more appealing for use. Nevertheless, it could be argued that the 15 items of the CAUSS are not all equality relevant in terms of the methodological quality of usability studies. The impact of using different methods to calculate the final score could be explored in future studies aiming at further refinement of the scale.

Reliability was assessed only by researchers involved in the development of the scale, which may have inflated the reliability results. In addition, we assessed interrater reliability only and did not test for test-retest reliability, which assesses the consistency of ratings for the same rater. Nevertheless, test-retest reliability is usually higher than interrater reliability, as interrater reliability refers to intersubject variability which is usually higher than intrasubject variability. The limited number of experts used to assess validity and the absence of other scales assessing the methodological quality of usability studies limit our ability to compare results. The future use of the developed scale to assess the methodological quality of other products/service will provide data on the reliability and validity of the scale. We encourage researchers to use the scale and to provide feedback.

In summary, the CAUSS scale, developed to assess the methodological quality of studies assessing usability, seems to be feasible to use and to have construct validity and interrater reliability. Further reliability, including test-retest reliability, and validity testing should be performed for different products and services, and the impact of using different methods to calculate the final score should also be explored.

## Appendix

Multimedia Appendix 1 Guide for the methodological quality assessment of studies that evaluate usability.

Multimedia Appendix 2 Methodological assessment of studies for the reliability and validity analysis of the scale. NA: not applicable; consensus score: final score after consensus; %: percentage out of maximum possible score.

## Abbreviations

CAUSS: Critical Assessment of Usability Studies Scale
eHealth: electronic health
ICC: intraclass correlation coefficient
mHealth: mobile health
