# Supplementary material for: A Scale to Assess the Methodological Quality of Studies Assessing Usability of Electronic Health Products and Services: Delphi Study Followed by Validity and Reliability Testing
Source: J Med Internet Res. 2019 Nov 15;21(11):e14829. doi: 10.2196/14829 (PMC6884719; doi:10.2196/14829)
Supplement: Multimedia Appendix 1 [file jmir_v21i11e14829_app1.pdf]

Guide for the methodological quality assessment of studies that evaluate usability

1. **Did the study use valid measurement instruments of usability (i.e. there is evidence that the instruments used assess usability)?**

This item is scored "**Yes**" if:

i) It is known that the instrument used was considered valid in previous studies and the study authors provide evidence of that (i.e., authors make a reference to previous studies); or

ii) It is known that the instrument used was considered valid in previous studies, but study authors do not provide evidence of that (i.e., authors make no reference to previous studies);

iii) Validity of instrument used was assessed as part of the study on usability;

iv) For qualitative data an effort was made to increase validity (using triangulation of methods and/or validation of the analysis and/or results by other researchers and by participants (Long & Johnson, 2000)).

This item is scored "**No**" if:

i) Instruments used are not considered valid or both valid and non-valid instruments are used;

ii) Authors provided insufficient information.

Note: The most common forms of validity testing for usability instruments are likely to be: i) construct validity (hypothesis testing) and/or ii) criterion validity. We recommended that it should be considered that there is evidence of validity in the following conditions: construct validity - the results are in accordance with pre-defined hypothesis; ii) criterion validity – correlation with a gold standard is ≥0.7 (Mokkink et al. 2018).

2. **Did the study use reliable measurement instruments of usability (i.e. there is evidence that the instruments used have similar results in repeated measures in similar circumstances)?**

This item is scored "**Yes**" if:

i) It is known that the instrument used was considered reliable in previous studies and the study authors provide evidence of that (i.e., authors make a reference to previous studies); or

ii) It is known that the instrument used was considered reliable in previous studies but the study authors do not provide evidence of that (i.e., authors make no reference to previous studies);

iii) Reliability of instrument used was assess as part of the study on usability;

iv) For qualitative data an effort was made to increase reliability (for example, using triangulation of researchers, providing the full description of the methods for data collection and analysis and accounting for personal and research method biases that may have influenced the findings).

This item is scored “**No**” if:

i) Instruments used are not considered reliable or both reliable and unreliable instruments are used;

ii) Authors provided insufficient information.

Note: The most common forms of reliability testing for usability instruments are likely to be: i) inter-rater reliability and/or ii) test-retest reliability using either an Intraclass Correlation Coefficient (ICC) or a Weighted K. We recommended that it should be considered that there is evidence of reliability if ICC and/or weighted K ≥0.7 (Mokkink et al. 2018).

3. Was there coherence between the procedures used to assess usability (e.g. instruments, context, …) and study aims?

This item is scored “**Yes**” if:

i) The procedures to assess the usability were chosen in accordance to the objectives of the study (for example, if the aim is to gather the subjective perception of participants, a more qualitative approach may be appropriate; if the aim is to collect data from a larger number of participants on a fully functional product a more quantitative assessment may be appropriate).

This item is scored “**No**” if:

i) Procedures to assess usability were not coherent with study aims; or

ii) Authors provided insufficient information.

4. **Did the study use procedures of assessment for usability that were adequate to the development stage of the product/service?**

This item is scored "**Yes**" if:

i) The procedures to assess usability were adequate to the stage of development of the product (for example, in the beginning of the product/service development, it is expected that usability assessments are performed laboratory environment and using experts; for a mature service/product it is expected that usability is assessed in real context with potential end users).

This item is scored "**No**" if:

i) Procedures to assess usability were not adequate to the stage of development of the product; or
ii) Authors provided insufficient information.

5. **Did the study use procedures of assessment for usability adequate to study participants' characteristics?**

This item is scored "**Yes**" if:

i) The procedures of assessment for usability was adequate to study participants' characteristics, particularly: age, cognitive function, educational level, clinical condition, technological literacy.

This item is scored "**No**" if:

i) Procedures to assess usability were not adequate to study participants' characteristics; or
ii) Authors provided insufficient information.

6. **Did the study employ triangulation of methods for the assessment of usability?**

This item is scored "**Yes**" if:

i) The study used a combination of at least two methods, one qualitative (for example, interviews) and the other quantitative (for example, questionnaires) to assess usability (across method triangulation); or

ii) The study used a combination of at least two methods, both qualitative or quantitative, to assess the usability (within-method triangulation).

This item is scored “**No**” if:

i) Only one method was used to assess usability; or

ii) Authors provided insufficient information.

7. Was the type of analysis adequate to the study’s aims and variables measurement scale?

This item is scored “**Yes**” if:

i) It is clear how the data was assessed, and the type of analyses was adequate (for example, content analysis for qualitative data and the appropriate statistical tests for quantitative data).

This item is scored “**No**” if:

i) The analysis was not appropriate (either a quantitative or a qualitative approach) or the statistical test used (quantitative data) was not the most appropriate.

ii) Authors provided insufficient information on the type of analysis performed (for example, they reported on using thematic analysis for qualitative data, but did not describe how the analysis was performed).

8. Was usability assessed using both potential users and experts?

This item is scored “**Yes**” if:

i) The study used participants recruited among potential users and participants who are considered experts.

This item is scored “**No**” if:

i) Only potential users or experts were used or none of them was used (for example, when a product is for a person with a clinical condition and it is tested in a healthy person);

ii) Authors provided insufficient information.

9. **Were participants who assessed the product/service usability representative of the experts' population and/or of the potential user's population?**

This item is scored "**Yes**" if:

i) Participants were representative of the population of experts and/or potential users. A minimal set of data should be given for experts (age, sex, area of expertise/professional occupation, years of practice, where they were recruited from) and users (age, sex, educational level, asymptomatic/clinical condition, where they were recruited from).

This item is scored "**No**" if:

i) Participants were not representative of the population of experts and/or potential users; or

ii) Authors provided insufficient information.

10. **Was the investigator that conducted usability assessments adequately trained?**

This item is scored "**Yes**" if:

i) The study refers that the investigator conducting the usability assessment had previous experience in the field (for example, had already conducted at least one usability assessment using the same method); or

ii) The study refers that the investigator conducting the usability assessment was trained by someone who has already performed usability assessments and give details on the training procedures (for example, time spent training or number of usability assessments performed).

This item is scored "**No**" if:

i) The study refers that the investigator conducting the usability assessment had no/insufficient previous experience;

ii) Authors provided insufficient information.

11. **Was the investigator that conducted usability assessments external to the process of product/service development?**

This item is scored "**Yes**" if:

i) The study refers that the investigator conducting the usability assessment was not involved in the development of the product.

This item is scored “**No**” if:

i) The study refers that the investigator conducting usability assessments was involved in the development of the product; or
ii) Authors provided insufficient information.

12. Was the usability assessment conducted in the real context or close to the real context where product/service is going to be used?*

This item is scored “**Yes**” if:

i) The study refers that the usability assessments were conducted in the context (or at least close to) in which the product is going to be used.

This item is scored “**No**” if:

i) The study refers that usability assessment s were conducted in laboratory or in a context different from the context where the product/service is going to be used; or
ii) Authors provided insufficient information.

13. Was the number of participants used to assess usability adequate (whether potential users or experts)?

This item is scored “**Yes**” if:

i) The study performed an a priori sample size calculation for quantitative assessments (for example, estimative of the sample size); or authors provide evidence that they reached the saturation point for qualitative studies;
ii) The study justifies sample size based on recommendations (for example, for formative evaluation a sample size of 5 to 10 participants are considered to be sufficient while for summative evaluations is necessary at least a sample size of 30 participants (Lewis, 2014)).

This item is scored “**No**” if:

i) The sample size used in the study was not justified or was considered small.

14. **Were the tasks that serve as the base for the usability assessment representative of the functionalities of the product/service?**

This item is scored "**Yes**" if:

i) The tasks performed to enable the assessment of usability were representative of the main functionalities of the product/service;

This item is scored "**No**" if:

i) The tasks used to assess the usability were not representative of the main functionalities (for example, the authors did not test the main functionalities, or few tasks were tested);
ii) Authors provided insufficient information.

15. **Was the usability assessment based on continuous and prolonged use of the product/service over time?***

This item is scored "**Yes**" if:

i) The product/service was use for several hours or days in the real context.

This item is scored "**No**" if:

i) The product/service was used for a very limited period of time in the presence of the investigator, usually with a pre-defined task to complete;
ii) Authors provided insufficient information.

*These items may be considered as non-applicable (N/A) depending on the phase of product development.

References:

Lewis, JR. Usability: Lessons Learned … and Yet to Be Learned. *International Journal of Human-Computer Interaction*, 2014:*30*(9), 663–684.

Long T, Johnson M. Rigour , reliability and validity in qualitative research. *Clinical Effectiveness in Nursing 2000:4*, 30–37.

Mokkink LB, Prinsen CAC, Patrick DL, Alonso J, Bouter LM, de Vet HCW, Terwee CB. COSMIN methodology for systematic reviews of Patient-Reported Outcome

Measures (PROMs). User Manual. 2018. https://www.cosmin.nl/
