# Supplementary material for: A Scale to Assess the Methodological Quality of Studies Assessing Usability of Electronic Health Products and Services: Delphi Study Followed by Validity and Reliability Testing
Source: J Med Internet Res. 2019 Nov 15;21(11):e14829. doi: 10.2196/14829 (PMC6884719; doi:10.2196/14829)
Supplement: Multimedia Appendix 2 [file jmir_v21i11e14829_app2.pdf]

Multimedia Appendix 2 – Methodological assessment of studies for the reliability and validity analysis of the scale (NA – not applicable; consensus score – final score after consensus; % – percentage out of maximum possible score).

Studies	Scale items																																													Consensus score	%
	1			2			3			4			5			6			7			8			9			10			11			12			13			14			15				
	A	B	C	A	B	C	A	B	C	A	B	C	A	B	C	A	B	C	A	B	C	A	B	C	A	B	C	A	B	C	A	B	C	A	B	C	A	B	C	A	B	C	A	B	C		
[20]	0	1	0	0	1	0	1	1	1	1	1	1	1	1	1	1	0	1	0	1	0	0	0	0	1	0	1	0	0	0	0	0	0	1	1	1	0	0	0	1	1	1	1	1	1	8	53.3
[21]	0	0	0	0	0	0	1	1	1	1	1	1	1	1	1	1	1	1	0	1	0	0	0	0	0	1	1	0	0	0	0	0	0	1	1	1	1	0	1	1	0	1	1	1	1	9	60,0
[22]	0	0	0	0	0	0	1	0	0	0	0	0	1	0	0	1	0	0	0	0	1	0	0	0	1	1	1	0	0	0	0	0	0	1	1	1	1	1	1	1	0	0	1	1	1	4	26.6
[23]	0	0	0	0	0	0	1	1	1	1	1	1	1	1	1	1	1	1	0	1	0	0	0	0	0	0	1	0	0	0	0	0	0	1	1	1	0	0	0	1	1	0	1	1	1	7	46.7
[24]	1	1	0	0	0	0	1	1	1	1	1	1	1	1	1	1	1	1	1	1	1	0	0	0	1	1	1	0	0	0	0	0	0	NA	NA	NA	1	1	1	1	1	1	NA	NA	NA	9	69.2
[25]	0	0	0	0	0	0	1	1	1	1	1	1	1	1	1	1	1	1	1	1	1	0	0	0	1	1	1	0	0	0	0	0	0	1	1	1	1	1	1	1	1	1	1	1	1	10	66.7
[26]	0	0	0	0	0	0	1	1	1	1	1	1	1	0	1	1	1	1	0	1	0	0	0	0	1	1	1	0	0	0	0	0	0	1	1	1	1	1	1	1	1	1	1	1	1	9	60.0
[27]	1	1	1	1	1	1	1	1	1	1	1	1	1	1	1	1	1	1	1	1	1	0	0	0	0	0	0	0	0	0	0	0	0	1	1	1	0	1	0	1	1	1	1	1	1	10	66.7
[28]	0	0	0	0	0	0	0	1	0	0	1	0	1	1	1	0	1	0	0	0	0	1	1	1	0	1	1	0	0	0	0	0	0	1	1	1	0	0	1	1	1	1	1	1	1	6	40.0
[29]	0	0	0	0	0	0	1	0	1	0	0	0	1	1	1	0	0	0	1	1	1	0	0	0	1	1	1	0	0	0	0	0	0	1	1	1	1	0	1	1	1	1	1	1	1	8	53.3
[30]	0	0	0	0	0	0	1	1	1	1	1	0	1	1	1	1	1	0	1	1	1	0	0	0	0	1	1	0	0	0	0	0	0	1	1	1	0	0	0	1	1	1	1	1	1	9	60.0
[31]	0	0	0	0	0	0	1	1	1	1	1	1	0	1	0	1	1	1	1	1	0	0	0	0	1	1	1	0	0	1	0	0	0	1	1	1	1	1	0	1	1	1	1	1	1	9	60.0
[32]	1	1	1	1	1	1	1	1	1	1	1	1	1	1	1	1	1	0	0	1	1	0	0	0	1	1	1	0	0	0	0	0	0	1	1	1	1	1	1	1	1	1	1	1	1	12	80.0
[33]	0	0	0	0	0	0	1	1	1	1	1	1	1	1	1	1	1	1	0	1	1	0	0	0	1	1	1	0	0	0	0	0	0	1	1	1	1	1	1	1	1	1	1	1	1	10	66.7
[34]	0	0	0	1	0	0	1	1	1	1	1	1	1	1	1	0	0	0	1	1	1	0	0	0	0	0	1	0	0	0	0	0	0	1	0	1	1	0	1	1	1	1	1	1	1	8	53.3
[35]	0	0	0	0	0	0	1	1	1	1	1	1	1	1	1	1	1	1	1	1	0	0	0	0	1	0	1	0	0	0	0	0	0	1	1	1	1	0	1	1	1	1	1	1	1	10	66.7
